# Recurrent Gastrointestinal Stromal Tumors in the Imatinib Mesylate Era: Treatment Strategies for an Incurable Disease

**DOI:** 10.1155/2017/8349090

**Published:** 2017-11-30

**Authors:** Rebecca M. Platoff, William F. Morano, Luiz Marconcini, Nicholas DeLeo, Beth L. Mapow, Michael Styler, Wilbur B. Bowne

**Affiliations:** ^1^Department of Surgery, Drexel University College of Medicine, Philadelphia, PA, USA; ^2^Department of Medicine, Division of Hematology/Oncology, Drexel University College of Medicine, Philadelphia, PA, USA; ^3^Department of Pathology and Laboratory Medicine, Drexel University College of Medicine, Philadelphia, PA, USA

## Abstract

**Introduction:**

Recurrence of gastrointestinal stromal tumors (GISTs) after surgical resection and imatinib mesylate (IM) adjuvant therapy poses a significant treatment challenge. We present the case of a patient who underwent surgical resection after recurrence and review the current literature regarding treatment.

**Case Presentation:**

A 58-year-old man with a large intra-abdominal jejunal GIST was treated with complete surgical resection followed by IM. The patient experienced disease recurrence 3.5 years later and underwent IM dose escalation and reresection.

**Conclusion:**

Current strategies to treat recurrent GIST include dose escalation, modifying adjuvant tyrosine kinase inhibitor therapy, and surgery. High-level evidence will be required to better define the combinatory roles of tyrosine kinase inhibitor therapy, guided by molecular profiling, and surgery in the management of recurrent GIST.

## 1. Introduction

Although rare, with an estimated incidence of 1.5 cases per 100,000 person years, gastrointestinal stromal tumors (GISTs) are the most common mesenchymal neoplasm of the gastrointestinal tract [1–5]. They arise from the interstitial cells of Cajal and most commonly occur in the stomach (50–60%), duodenum and small bowel (20–35%), rectum (5%), esophagus (2%), and rarely in the omentum, mesentery, and retroperitoneum [1, 6, 7].

A breakthrough in the management of GISTs was the development of tyrosine kinase inhibitors (TKIs), most notably imatinib mesylate (IM), which targets a mutation in *c-Kit*, a gene encoding a tyrosine kinase receptor and found in 80-90% of patients with GIST [1, 6, 8–12]. Before the development of targeted therapy, greater than 50% recurred within two years of surgery [1, 6, 10, 13]. IM is recommended by the FDA in the adjuvant setting for intermediate/high-risk disease as a result of the Z9001 trial and has been used in the neoadjuvant setting for potentially resectable disease [9, 14, 15]. DeMatteo et al. demonstrated in this randomized controlled trial that one year of IM improved recurrence-free survival as compared to placebo, regardless of tumor size [9]. In 2012, Joensuu et al. further showed that three years of adjuvant imatinib conferred an overall survival advantage compared with one year of treatment [16]. This therapy has become the mainstay of adjuvant treatment for intermediate/high-risk GISTs [6, 17].

Despite remarkable improvements, management of recurrent disease remains largely undefined, in particular the role of surgical resection in recurrent disease. We present a case of recurrent GIST managed surgically after progression on adjuvant TKI therapy and review the current literature regarding management strategies for recurrent GIST.

## 2. Case Report

**Figure 1:**
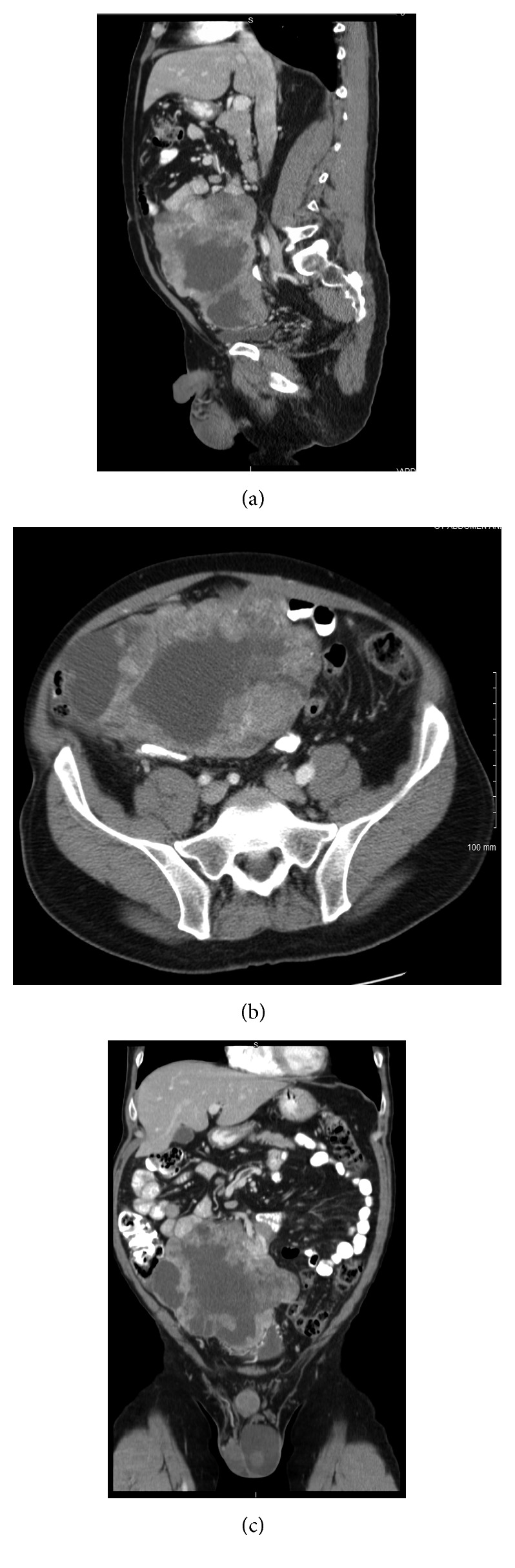
Sagittal (a), axial (b), and coronal (c) images of CT with IV contrast showing the large, lobulated primary mass that was discovered in January 2013.

A 58-year-old man presented to his primary care physician with vague abdominal pain, constipation, and one year of urinary hesitancy. Abdominopelvic CT scan revealed a complex, lobulated, enhancing, intraperitoneal mass measuring 18 × 19 × 10 cm, extending from the umbilicus to the level of the superior acetabulum (Figure 1). He underwent resection of the mass, including small bowel and partial bladder resection in January 2013. Pathology confirmed complete resection (R0) of high-grade GIST originating from the proximal jejunum, stage IIIB (pT4a, pNx). The tumor was spindle-cell subtype, with the mitotic rate of 8/10 HPF, and necrosis. Immunohistochemistry (IHC) showed membrane positivity for CD117, beta-catenin, vimentin, and smooth muscle actin (SMA). Mutational analysis demonstrated no mutations in the *c-Kit* proto-oncogene or platelet-derived growth factor receptor alpha (*PDGFRA*). Risk of recurrence was determined to be 90% [18–23]. He began imatinib mesylate 400 mg daily postoperatively. No evidence of tumor recurrence was detected over three years postoperatively. The patient tolerated imatinib well, except for mild diarrhea (grade 1 CTCAE) [24].

**Figure 2:**
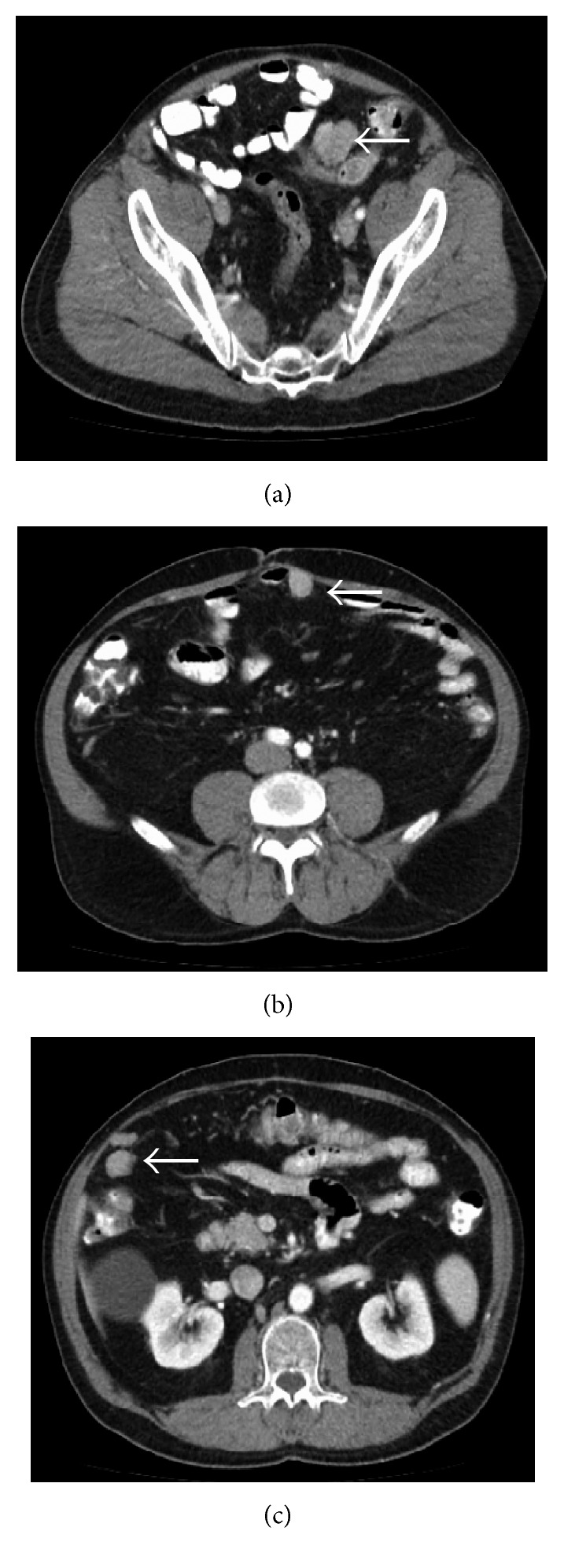
Axial CT images demonstrating recurrent lesions (white arrows) in the left lower quadrant (a), adherent to the anterior abdominal wall (b), and in the right upper quadrant (c).

**Figure 3:**
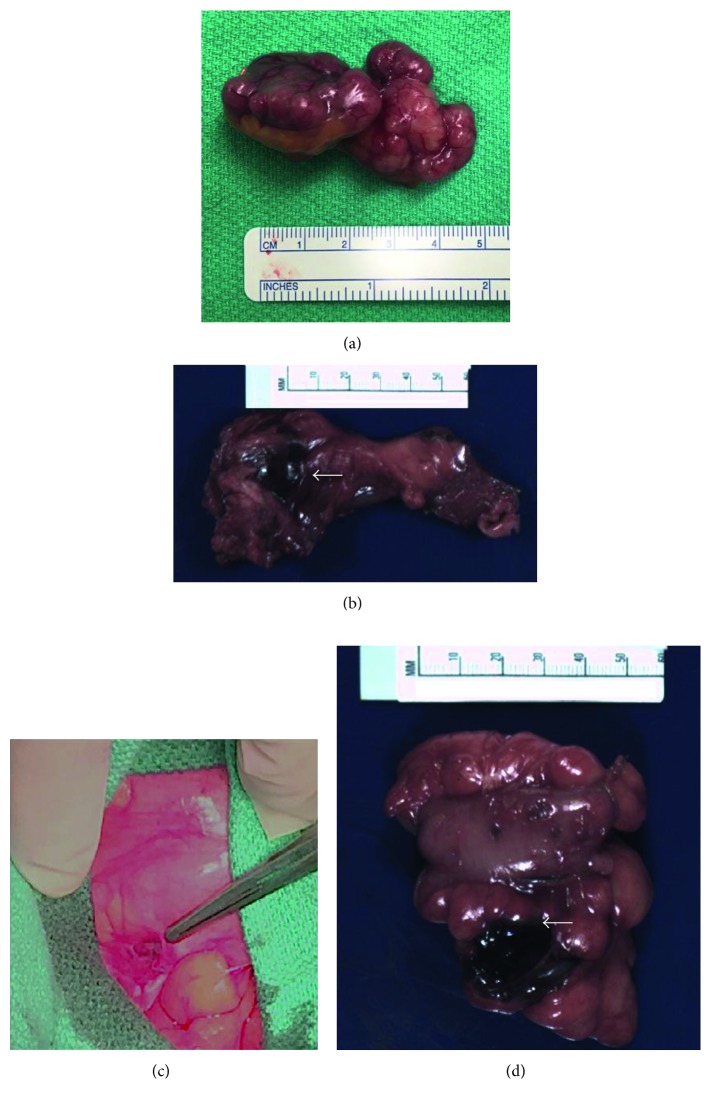
(a) Gross specimen of right upper quadrant recurrent GIST lesion. (b) Anterior abdominal wall mass (white arrow) adherent to the resected loop of the small intestine. (c) Small nodule within the mesentery discovered on diagnostic laparoscopy and resected. (d) Gross specimen demonstrating necrotic sigmoid lesion (white arrow) adherent to resected sigmoid.

In May 2016, surveillance CT revealed a 3.5 × 2.8 cm left lower quadrant mass abutting the sigmoid colon (Figure 2). IM dosage was empirically increased from 400 mg to 800 mg daily, but repeat imaging in July 2016 showed disease progression. The left lower quadrant lesion had grown in size to 4.0 × 3.3 × 3.2 cm, with extrinsic compression on the sigmoid colon, with a 2.4 × 2.1 × 2.5 cm periumbilical lesion. Additionally, a new right upper quadrant lesion was noted, approximately 5.3 × 3.7 × 2.1 cm. In late July 2016, diagnostic laparoscopy was performed (detecting a right lower quadrant peritoneal nodule), followed by laparotomy, small bowel resection, resection of right upper quadrant lesion, sigmoidectomy, and resection of right lower quadrant peritoneal nodule (Figure 3). The pathology revealed high-grade GIST with negative margins and absence of *c-Kit* mutation. Molecular profiling and next-generation sequencing (NGS) of the recurrent disease indicated susceptibility to sunitinib based on the presence of wild-type (WT) *c-Kit*. Accordingly, the patient was switched from imatinib to sunitinib. Follow-up CT in May 2017 showed no signs of tumor recurrence, with patient follow-up at three-month intervals [14].

## 3. Discussion

In the case of primary GIST, surgery remains the definitive therapy for patients with low- and intermediate-risk disease [25]. For patients with high-risk disease (defined by the NIH Consensus Criteria as [1] size >10 cm, [2] mitotic rate > 10/50 hpf field or [3] mitotic rate  > 5/50 hpf and tumor size > 5 cm, or [4] tumor rupture spontaneously or at surgery), adjuvant TKI therapy has been shown to add significant survival benefit [9, 26].

The Z9001 Trial revolutionized the treatment of GIST, demonstrating improvement in 1-year recurrence-free survival of 98% versus 83% in treatment and placebo groups, respectively [9]. Thereafter, the Scandinavian Sarcoma Group (SSG) trial, comparing 1 and 3 years of imatinib therapy, showed improved 5-year recurrence-free survival of 47.9% and 65.6%, respectively [16]. Of note, approximately 15% of GISTs have no detectable *c-Kit* or *PDGFRA* mutation [27]. The benefit from adjuvant imatinib is minimal in *c-Kit/PDGFRA*-WT patients. Specifically, in the study by Corless et al., imatinib was associated with higher recurrence-free survival versus placebo in patients with *c-Kit* exon 11 deletions but was not significantly associated with *PDGFRA* mutation or wild-type tumors [28]. Thus, risk of recurrence is higher, and treatment with imatinib is debated [29]. Nevertheless, NCCN recommendations suggest continued use of adjuvant imatinib therapy for these patients.

GIST recurrence in the IM era is largely considered incurable, and treatment strategies are aimed at delaying progression [6, 16]. Despite response to TKI therapy, many patients with high-risk GIST eventually develop recurrent disease [6]. In the SSG study, 65.6% of those who completed 3 years of adjuvant imatinib were alive without recurrence 5 years after study entry. However, 34.4% of those treated experienced recurrence requiring further management [16].  Table 1 highlights current institutional studies demonstrating benefit of TKI therapy for recurrent GIST. Treatment options are to initially escalate TKI dose or switch to a second-line drug, typically sunitinib malate [6, 13]. Imatinib can be increased from 400 mg to 800 mg daily, with an approximate 30% response rate in patients with KIT exon 9 mutations and acceptable toxicity profile [6, 31]. While *c-Kit* exon 11 mutations tend to have a higher response to imatinib, primary resistance in the first 6 months of treatment can occur with *c-Kit* exon 9, exon 18, and *PDGFRA* mutations [35–37]. Secondary resistance after six months can be observed with acquisition of new KIT kinase mutations such as in *c-Kit* exon 17 or *c-Kit* kinase domain 1 [38–40].

Sunitinib targets *c-Kit* and PDGFR-alpha and -beta receptors, among others [6]. In our patient, after resection of recurrence, NGS demonstrated a WT *c-Kit*, signaling potential benefit with sunitinib. Clinical benefit (partial response or stable disease for greater than or equal to 6 months) with sunitinib was observed with progression-free and overall survival in imatinib-resistant GIST [41]. In patients with WT *c-Kit*, Heinrich et al. showed a median progression-free survival of 19 months for patients treated with sunitinib after progression on imatinib versus 5.1 months for those with exon 11 mutations (*p*=0.03) [41]. Similarly, a study by Demetri et al. showed improved median time to tumor progression for sunitinib versus placebo of approximately 27 weeks versus 6 weeks [30]. Subsequent progression from second-line therapy can then be treated with regorafenib, an oral multikinase inhibitor with increased progression-free survival but not overall survival compared to placebo [33].

A key principle in treatment of recurrent and/or metastatic GIST is to continue imatinib or second-line therapy indefinitely, as it has been shown that patients who discontinue therapy have higher rates of disease progression [6]. Moreover, recent studies have found strong linear correlations between survival time and duration of TKI therapy after diagnosis of recurrence/metastasis [1, 13, 26, 42]. NGS of the 592 genes most commonly associated with cancer, should expand our understanding of clonal evolution and pathogenesis of disease (high-risk primary and recurrence).

Another avenue in the early phase of exploration is treatment with immunotherapy. Seifert et al. analyzed 85 patients with GIST to determine expression of immune checkpoint molecules and the effects of combination imatinib and PD-1/PD-L1 blockade in KitV558Δ/+ mice that develop GIST. The PD-1 inhibitory receptors were upregulated on tumor-infiltrating T-cells as compared to T-cells from matched blood. PD-1 and PD-L1 blockade in vivo had no efficacy alone but enhanced the antitumor effects of imatinib by increasing T-cell effector function [34].

In addition to TKIs, surgery remains an important consideration in the management of recurrent GIST (Table 2). GISTs may follow disease-specific patterns that make recurrence amenable to resection [10, 42]. To demonstrate the benefit of surgery itself, studies have focused on its role in GIST recurrence regardless of patients' TKI use. A 2015 retrospective review of 186 patients showed that surgery for resectable, recurrent GIST was associated with increased overall survival compared to patients with resectable disease on TKI therapy alone [13]. In this study, 56 patients experienced recurrence, 30 with resectable disease. Twenty-four of those patients underwent upfront surgery (of which 18 received imatinib postoperatively) and 6 opted for nonoperative management. Their results showed a 1-year survival of 100% for those who underwent surgery compared to 50% with medical management alone, with 3-year survival rates of 80% versus 50% (*p*=0.04), respectively. While surgery alone improved survival over TKI therapy only, their data also demonstrated a median disease-free survival of 2.9 years for patients who underwent surgery while on imatinib, as compared to 1.4 years after surgery alone. This study established the benefit of upfront surgery for GIST recurrence regardless of response to adjuvant TKI therapy, while also highlighting the combinatory effect of these two treatment strategies. The authors suggest that in patients with resectable, recurrent disease, complete resection of recurrent GIST may eliminate possible mutant strains, avoiding the need for escalation of TKI dosage [13, 15].

Further studies into surgical management of recurrent GIST have shown optimal recurrence-free and overall survival if patients are responding to TKI therapy at the time of surgery. Winer and Raut recommend that imatinib therapy commence prior to surgery, and surgeons should wait a minimum of six months before proceeding with resection [6]. Furthermore, retrospective reviews from the Istituto Nazionale dei Tumori, Memorial Sloan Kettering Cancer Center, and Brigham and Women's Hospital/Dana-Farber Cancer Center demonstrated that these patients benefit most when disease progression has stabilized on imatinib, or less commonly on sunitinib [44–46]. Similarly, Chang et al. showed that timing of surgery relative to TKI therapy may contribute to outcome in a review of 182 patients with advanced/recurrent GIST [17]. In this study, 76 patients undergoing cytoreductive surgery were divided into an “early” group (prior to imatinib use, *n* = 54) and a “late” group (after imatinib use, *n* = 22). Those in the late surgery group had a higher rate of R0 resection (59.1% versus 31.6%, *p*=0.02), higher complete and partial response rates (100% versus 79.6%, *p*=0.02), and improved trend in overall survival. The authors imply that as surgery reduces tumor burden, this may delay time to development of secondary resistance, and offers a survival benefit when imatinib therapy is initiated prior to surgery [17].

The quality of resection for GIST recurrence has been found to play a pivotal role in survival [6, 25]. A 2016 study by Sato et al., analyzing data from forty Japanese institutions, showed that overall survival is significantly improved with R0/R1 resection [25]. Of the 93 included patients who experienced recurrence, 50 underwent surgery. Those with R0/R1 resection (*n* = 34) had significantly higher 5-year overall survival as compared to R2 resection (*n* = 13) (82.2% versus 47.0%, *p*=0.018). Notably, the authors found a survival benefit from curative resection but reduced 5-year overall survival for R2 resection as compared to TKI therapy only (47% versus 60.2%). Their study concluded that surgical intervention should be reserved only for patients with possibility of achieving R0/R1 resection, 6–12 months after initiation of imatinib therapy. Importantly, R0/R1 resection of residual disease had a benefit when the number of metastatic lesions was less than 4, total tumor size was less than 100 cm, and disease remained stable or responsive to TKI therapy [25].

Laparoscopy has become an important consideration in the management of primary GISTs, both for diagnostic and therapeutic purposes, yet literature is sparse regarding its contribution for recurrence. Currently, NCCN guidelines support the use of a laparoscopic approach for resection of GIST in anatomically favorable locations (anterior wall of the stomach, ileum, and jejunum), while also noting that its use may expand after further studies due to the decreased short-term morbidity of this approach [14]. Likewise, diagnostic laparoscopy may be a valuable adjunct when approaching these patients with recurrent or metastatic disease to determine resectability or detect lesions not visualized on imaging.

CT remains the imaging modality of choice for surveillance and selection of patients with recurrence that may be candidates for surgical resection. This allows for monitoring disease progression via a change in size, development of new lesions, or alteration in density on CT demonstrating a response to TKI therapy. Tumor treatment-response, or lack thereof, will help guide whether surgical resection of recurrent disease is appropriate [47]. However, in our patient, laparoscopy allowed for detection of a subradiographic lesion not previously visualized on CT, facilitating complete resection in this patient with high-grade, recurrent GIST.

Paucity of high-level evidence investigating the management of recurrent GIST calls for prospective, randomized controlled studies to evaluate the benefit of surgery compared with TKI therapy alone. The difficulty with conducting such trials is elaborated by Du et al. who explain that in their experience, both patients and surgeons are resistant to the idea that a computer algorithm is the decision maker for randomizing an intervention as major as surgery. Their prospective, randomized trial comparing surgery and IM therapy for recurrent/metastatic GIST enrolled 41 patients, far short of the planned 210. This study investigated only patients with recurrence and continued response to IM and showed that median overall survival was prolonged in patients who underwent surgery. While their findings were encouraging, they lacked statistical significance due to poor patient accrual [43].

## 4. Conclusion

Recent literature demonstrates a survival benefit with surgical intervention in patients with recurrent GISTs. Factors that may improve survival after surgical management of recurrent GIST include quality of resection, limited burden of disease, and response to TKI therapy. If recurrence develops while on TKI therapy, progression-free survival may be improved with dose escalation or next-generation TKIs. Further studies are now needed to elucidate the relative importance of these factors, particularly their impact on patient survival, such as ours, who progressed on TKI therapy, but otherwise had resectable disease with few metastases and optimal performance status. Current literature offers insight into the role of surgery for improving survival in patients with recurrent GIST, with the most significant deficit being whether surgery can provide survival benefit to patients no longer responding to TKI therapy. Clearly, the roles of TKIs and surgery for improving survival in patients with recurrent GIST are not mutually exclusive. Prospective, randomized trials will be required to develop treatment algorithms to delineate combinatory roles of TKIs, guided by molecular profiling, and surgery in the management of recurrent GIST.

## Figures and Tables

**Table 1:** Institutional studies demonstrating benefit of TKI therapy for recurrent GIST.

	Study design	Number of patients	Primary endpoint	Main findings
Demetri et al. [30]	Randomized, double-blind, placebo-controlled, multicenter, international trial comparing sunitinib versus placebo after imatinib failure	321 (207 sunitinib versus 105 placebo patients)	Tumor progression	Median time to tumor progression: 27.3 weeks in patients receiving sunitinib versus 6.4 weeks with placebo

MetaGIST [31]	Analysis of two large, randomized, cooperative group studies comparing two doses of IM (400 mg daily versus twice daily) in 1640 patients with advanced GISTs	1640 (data analysis after 344 and 321 cases of progression or death in each study)	PFS and OS	High-dose imatinib 800 mg daily improved PFS but not OS compared to imatinib 400 mg daily

Reichardt et al. [32]	Randomized phase III open-label trial comparing nilotinib versus best supportive care with advanced GIST following prior imatinib/sunitinib failure	248 (2 : 1 randomization nilotinib or best supportive care)	PFS, OS	Subset analysis of patients with one prior regimen each of imatinib and sunitinib showed significant increase in median OS in favor of nilotinib versus best supportive care

Demetri et al. [33]	Randomized, double-blinded, placebo-controlled, multicenter, international trial comparing regorafenib versus placebo after imatinib/sunitinib failure	199 (133 regorafenib versus 66 placebo patients)	PFS	Median PFS 4.8 months for regorafenib versus 0.9 months for placebo

Seifert et al. [34]	Analysis of 85 patients with GISTs to determine expression of immune checkpoint molecules and effects of combination IM + PD-1/PD-L1 blockade in murine GISTs	85 (blood samples from patients with GISTs)	PD-1 receptor expression in T-cells of human GISTs	The PD-1 inhibitory receptors were upregulated on tumor-infiltrating T-cells compared with T-cells from matched blood
			T-cell function in mice with GISTs treated with IM and PD-1/PD-L1 inhibitor	PD-1 expression on T-cells was highest in IM-treated human GISTs
				PD-1/PD-L1 blockade in vivo had no efficacy alone but enhanced antitumor effects of IM by increasing T-cell effector function

IM = imatinib mesylate, PFS = progression-free survival, OS = overall survival.

**Table 2:** Institutional studies demonstrating benefit of surgery for recurrent GIST.

	Study design	Number of patients	Primary endpoint	R0 resection	Main findings
Bischof et al. [1]	Multi-institutional retrospective cohort	158 (87 locally advanced, 71 recurrent/metastatic)	RFS, OS	69% (recurrent/metastatic) versus 87.4% (locally advanced)	TKI-sensitive recurrent/metastatic disease—improved RFS, OS after surgery

Du et al. [43]	Phase III multicenter trial for recurrent/metastatic on IM +/− surgery for residual disease	41 (19 IM + surgery, 22 IM alone)	PFS	73.6%	Trend towards improved PFS in surgery group

Tan et al. [13]	Retrospective cohort—upfront surgery versus TKI for recurrence	186 (56 recurrent—30 resectable, 24 underwent surgery for recurrence)	DFS, OS	75% (18 of 24) in upfront surgery group	Improved OS and DFS with surgery

Chang et al. [17]	Prospectively collected retrospective review—imatinib + surgery (early versus late groups) versus IM only	182 (89 metastatic, 93 recurrent, 76 underwent surgery)	Clinical response, PFS, OS	31.5% (early surgery) versus 59.1% (late surgery)	Improved CR, PR, PFS, OS in early surgery group; improved CR, PR, OS in late surgery group

Sato et al. [25]	Retrospective cohort comparing IM + surgery to surgery only	737 (93 recurrent/metastatic—50 surgery + TKI therapy, 43 TKI therapy alone)	DFI, OS	58% (29 of 50)	Improved survival from surgery + TKI after complete resection, response to TKI, < 4 metastatic lesions, lesions < 100 mm total

TKI = tyrosine kinase inhibitor, IM = imatinib mesylate, OS = overall survival, DFS = disease-free survival, PFS = progression-free survival, CR = complete response, PR = partial response, RFS = recurrence-free survival, DFI = disease-free interval.
